# A Review of Novel Strategies for Human Periodontal Ligament Stem Cell Ex Vivo Expansion: Are They an Evidence-Based Promise for Regenerative Periodontal Therapy?

**DOI:** 10.3390/ijms24097798

**Published:** 2023-04-25

**Authors:** Anna Di Vito, Jessica Bria, Alessandro Antonelli, Maria Mesuraca, Tullio Barni, Amerigo Giudice, Emanuela Chiarella

**Affiliations:** 1 Department of Experimental and Clinical Medicine, University Magna Græcia of Catanzaro, 88100 Catanzaro, Italy; divito@unicz.it (A.D.V.); jessica.bria@studenti.unicz.it (J.B.); mes@unicz.it (M.M.); barni@unicz.it (T.B.); 2 Department of Health Science, University Magna Græcia of Catanzaro, 88100 Catanzaro, Italy; Antonellicz@gmail.com (A.A.); a.giudice@unicz.it (A.G.)

**Keywords:** hPDLSCs, in vitro proliferation, periodontal regeneration, drugs, chemical, cytokines, platelet-rich plasma, allogenic human serum

## Abstract

Periodontitis is a gingiva disease sustained by microbially associated and host-mediated inflammation that results in the loss of the connective periodontal tissues, including periodontal ligament and alveolar bone. Symptoms include swollen gingiva, tooth loss and, ultimately, ineffective mastication. Clinicians utilize regenerative techniques to rebuild and recover damaged periodontal tissues, especially in advanced periodontitis. Human periodontal ligament stem cells (hPDLSCs) are considered an appealing source of stem cells for regenerative therapy in periodontium. hPDLSCs manifest the main properties of mesenchymal stem cells, including the ability to self-renew and to differentiate in mesodermal cells. Significant progress has been made for clinical application of hPDLSCs; nevertheless, some problems remain, including the small number of cells isolated from each sample. In recent decades, hPDLSC ex vivo expansion and differentiation have been improved by modifying cell culture conditions, especially with the supplementation of cytokines’ or growth factors’ mix, chemicals, and natural compounds, or by using the decellularized extracellular matrix. Here, we analyzed the changes in stemness properties and differentiation potential of hPDLSCs when culturing in alternative media. In addition, we focused on the possibility of replacing FBS with human emoderivates to minimize the risks of xenoimmunization or zoonotic transmission when cells are expanded for therapeutic purposes.

## 1. hPDLSCs as Therapeutic Tool: Current Status

Periodontitis is the most common chronic inflammatory oral disease characterized by progressive destruction of periodontal ligament (PDL) and alveolar bone resorption [1].

PDL is a fibrous connective tissue that connects the cementum and the inner wall of the alveolar bone socket to support teeth in situ. PDL exhibits dynamic tensile properties important for promoting alveolar bone remodeling throughout adulthood, thus playing a key role in maintaining tissue homoeostasis and structural integrity [2].

The establishment of an inflammatory environment in periodontitis is accompanied by the loss of human Periodontal Ligament Stem Cells (hPDLSCs) and the rapid disruption of PDL due to an imbalance between catabolic and anabolic tissue changes associated with tensile and compressive strains, respectively. Similar events were reported in alveolar bone, where the disorganized remodeling ultimately accounts for bone resorption [3]. 

The primary cause of periodontitis is the accumulation of plaque on the teeth and gingiva in the oral cavity. Dental plaque is a soft, sticky biofilm composed of oral bacteria, mucus and sugars that usually forms as a result of poor personal oral hygiene [1].

Other well-established risk factors for periodontal disease are associated with tobacco smoking, unmanaged diabetes, and a poor diet, especially one deficient in calcium, vitamin C and group B vitamins. In turn, periodontitis has been linked to reduced food intake and malnutrition, which account for declined functional ability such as immune dysfunction. The evident strong correlation between the spread of periodontitis and socioeconomic conditions in the world underlies the tight interconnection between human health and the economy [4,5]. 

The pathogenesis of periodontitis has been well described. After dysregulation of dental biofilm, an excessive immune response seems to be mandatory to trigger apical migration of the epithelium and periodontal pocket formation. The accumulation of bacteria in the periodontal socket accounts for the release of a great number of proinflammatory factors, such as fimbriae, lectin-type adhesins, LPS, hemagglutinins, proteinases and other enzymes as well as outer membrane vesicles that account for the creation of a proinflammatory microenvironment. Therefore, the pocket epithelium also contributes to the maintenance of proinflammatory conditions. The resulting inflammatory microenvironment accounts for massive neutrophil recruitment and the release of extremely high quantities of proinflammatory cytokines able to damage host tissues, including soft tissue and, in the most serious cases, the bone that supports the teeth [6].

Several symptoms are common to dental plaque-induced inflammatory conditions, including gingiva redness and bleeding, pus between the teeth and the gingiva, bad breath and swelling, pain while chewing, and loosened teeth. However, the type and severity of symptoms depend on the stage and grade of the disease according to the most recent guidelines defined during the 2017 World Workshop on the Classification of Periodontal and Peri-Implant Diseases and Conditions [7]. Staging (I–IV) defines the progressive levels of severity as well as the extent and complexity of the management required based on different factors, including clinical attachment loss, radiographic bone loss and tooth loss. Grading (A–C) measures the progression rate of the disease based on patients’ associated risk factors and predicts treatment outcomes and possible adverse effects on the general health of the patient exacerbated by the disease or its treatment [8,9]. Recently, stage IV has been introduced by Herrera et al., indicating that periodontal treatment alone will not be enough, and periodontitis is weighed down by anatomical and functional sequelae deriving from tooth and periodontal attachment loss. In these case, additional multidisciplinary interventions are required [9].

In the early stages, the periodontitis treatment involves professional cleaning and good oral hygiene, as well as the treatment of all risk factors. When periodontitis is persistent and rapidly progresses, antibiotics are recommended both locally and systemically, according to periodontitis severity [10]. In patients with aggressive periodontitis, periodontal surgery may be required for the reduction of periodontal pocket depth and/or the replacement of damaged and lost tissues. Among the different strategies aimed to achieve the simultaneous regeneration of cementum, PDL and alveolar bone, bone transplantation, allogeneic materials, guided tissue regeneration (GTR), and stem cell-based tissue engineering show solid evidence for clinical application in human periodontal defects [11,12,13].

Interestingly, stem cell-based tissue engineering stands as a promising treatment strategy for the regeneration of all periodontal tissues as a functional unit [14]. 

Stem cells populating PDL reside in the perivascular space of the periodontium and contain 95% of mesenchymal stem cells and 5% of neural crest stem cells, according to their embryonic origin from ectomesenchyme [15,16]. Nowadays, mesenchymal stem cells are revolutionizing the field of oral diseases, representing a potential tool for periodontal regeneration [17]. 

Like MSCs isolated from bone marrow, cord blood, adipose tissue, nasal mucosa, dental pulp and apical papilla, hPDLSCs have the ability to grow clonally. Furthermore, when hPDLSCs are cultured, they showed positive expression for the mesenchymal markers CD73, CD90, CD105, CD29, CD44, CD146 and CD166, and negative expression of monocyte (CD14) and hematopoietic (CD34, and CD45) markers, endothelial marker CD31 and adhesion markers CD66, CD144, and CD171. This immunophenotype profile, recognized as a hallmark of multipotency, highlights the differentiation potential of hPDLSCs into various lineages such as chondrocytes, neural cells, cardiomyocytes, and mostly osteoblasts and cementoblasts which are implied in sustaining the formation of new bone [18,19,20]. In addition, hPDLSCs exert a strong paracrine capacity by releasing several angiogenic, mitogenic, antiapoptotic, anti-inflammatory and antioxidative factors, which together are proposed as the main mechanisms in regulating tissue repair upon MSC transplantation [16,21,22,23,24,25].

Recently, significant advances have been made in the field of hPDLSC-based therapy. From their discovery, various attempts to induce periodontal regeneration have been made by combining hPDLSCs with scaffolds or membranes and with signaling molecules [26,27]. Cell-sheet technologies have also been developed via approaches that can involve or not the use of scaffolds aimed at supporting cell attachment, proliferation and 3D tissue organization [28,29]. 

Despite the development of different therapeutic approaches involving hPDLSCs, oral MSCs-based therapy is still in its infancy and displays many limitations. After administration, MSCs migrate to the focus of injury via the “homing” mechanism and then differentiate into multiple cell types and/or secrete bioactive factors. The lack of solid knowledge about the mechanisms by which MSCs exert their therapeutic action represents the first important limitation. Second, hPDLSC samples often require in vitro expansion to obtain a cell population suitable for infusion or implantation. The low number of immature cells that can be obtained by a single sample, as well as the loss of stemness when they are maintained in culture for a long time, represents a major limitation in clinical application. In general, the stemness of MSCs is maintained up to 6 passages and significantly decreases after 10 passages due to senescence-related phenomena [22,30]. Third, the protocols for cell isolation and culturing, as well as for cryopreservation, require further standardization. 

The evidence that most of the therapeutic effects of MSCs are related to the release of molecules with paracrine and anti-inflammatory effects point the attention to the development of strategies aimed at ameliorating paracrine action of MSCs. Indeed, the use of hPDLSC secretome could overcome some major challenges and controversies relating to MSCs’ application, such as a potential ectopic differentiation and transformation in cancer cells [23]. Nevertheless, secretome-based therapy still requires cell expansion in order to obtain sufficient quantity of bioactive molecules to be used for medical purpose. 

Based on the above considerations, we can conclude that many concerns still arise regarding the use of hPDLSCs in clinical trials. It’s remarkable the approach proposed by Vandana et al., which consists of the direct application of autologous hPDLSCs for intrabony periodontal defects, bypassing ex vivo culture [31,32]. From their results, it appeared that hPDLSCs contributed to defect regeneration; however, no evidence was found regarding the number and viability of cells transplanted or effective cell integration. Therefore, further investigations are required to validate this technique. 

The large-scale expansion of MSCs required for cell therapies remains a great challenge. In the last decades, new culture media formulations obtained via the addition of specific molecules or serums were proposed to expand hPDLSCs ex vivo without altering stemness and differentiation potential. Here, we critically discuss the hPDLSC culture media formulations proposed over the past 15 years, weighing the pros and cons.

## 2. Pharmacological Approaches to Improve hPDLSC Expansion

The success of cell therapy for periodontal repair and regeneration is closely related to long-term hPDLSC in vitro expansion which results in cellular senescence and autophagy impairment, especially during later passaging. In standard culture conditions, senescence is evidenced by a decrease in proliferation and osteogenic differentiation as well as in the enhancement of senescence-associated protein levels, including p16, p21, p53, and γ-H2AX, and senescence-associated β -galactosidase activity, which together are considered hallmarks of DNA damage [33,34,35]. 

Several strategies, such as genetic modifications and physiological and pharmacological preconditioning, have proven effective for hPDLSC expansion, allowing a sufficiently high number of cells for therapeutic use in fewer passages to be obtained. Most of them also preserve their therapeutic potential, guaranteeing both the osteogenic differentiation program and the therapeutic action of their secretome.

Cell culture media development and optimization are critical to promote hPDLSC viability and robust growth and to maintain cell phenotype. Today, we tend to enrich media formulations with several cytokines’ and growth factors’ mix, chemicals, natural components, amino acids or vitamins to improve stem cell clonogenicity and differentiation potential [36]. In addition, new alternative and interesting methods for ex vivo expansion and osteoblastic differentiation of hPDLSCs involve the plating of these multipotent stem cells on the decellularized extracellular matrix (ECM).

### 2.1. Cytokines and Growth Factors

In the last 15 years, many cytokines and growth factors have been employed as culture media additives for in vitro cell growth and differentiation. Among them, the addition of Fibroblast growth factor 2 (FGF-2), VEGF, EGF, IGF-1, BMP-2, BMP-4, EPO, SDF-1, PTH, Neuregulin-1 and ADAM28, alone or in different combinations, showed interesting results. Altogether, these approaches provided us with the first milestones in the research aimed at defining the best concentration and exposure times of each cytokine. The cytokines and growth factors used as additives are listed in Table 1.

FGF-2 is a cytokine widely recognized as potent stimulator of MSCs proliferation and preservation in a pluripotent state; however, it has been reported to suppress in vitro mineralization of hPDLSCs in a dose-dependent manner (up to 20 ng/mL) [37]. To overcome these limitations, the combination of FGF-2 and A83-01 proved to be a good strategy for improving the biological behavior of hPDLSCs when expanded in 10% FBS alpha-MEM culture medium. A83-01 is a selective inhibitor of TGF-βRs, which contribute to stem cell expansion by inhibiting SMAD2 phosphorylation. After 48 h of FGF-2 (10 ng/mL) and A83-01 (5 μM) preconditioning culture, reinforced hPDLSC expansion associated with a significant reduction in cell apoptosis, a higher expression of stemness markers, increased terminal osteogenic differentiation and cytokines secretion compared to FGF-2 alone was documented [37].

Similarly, the treatment of hPDLSCs with 25 ng/mL of VEGF enhanced the effect of FGF-2 (25 ng/mL) on cell proliferation but could not antagonize the strong inhibitory effect of FGF-2 during osteogenic differentiation. The supplementation with VEGF alone has positive effects on odonto-/osteogenic differentiation in vitro, upregulating the mRNA level of osteoblastic-related genes and on the formation of new mineralized structures in vivo [38]. A new protocol involving the sequential application of 25 ng/mL FGF-2 for 3 days followed by the integration of 50 ng/mL BMP-2 for another 9, 18 and 25 days proved particularly effective in promoting osteogenic differentiation of hPDLSCs. This strategy would provide an attractive new approach for ex vivo expansion of stem cells for periodontal regeneration without affecting osteoblastic differentiation processes [39].

A similar approach has been experimented with by Hyun et al. (2017). They investigated the effects of FGF-2 alone, BMP2/4 alone and FGF-2 in combination with BMP2/4. For cotreatment of FGF-2 and BMPs, cells were pretreated with FGF-2 for 2 days and then exposed to FGF-2 and BMPs simultaneously. As expected, hPDLSC treatment with BMP-2 or BMP-4 alone increased osteogenesis, while exposure to FGF-2 significantly reduced their action. However, the authors did not analyze the effects of cotreatment, so further analyses still need to confirm or exclude a synergistic effect of BMPs with the positive action of FGF-2 on cell proliferation [71]. 

The ex vivo expansion of hPDLSCs could be effectively supported by the formulation of culture media with a balanced cytokine mix. In this scenario, both doses and exposure times of cytokines significantly affect the success of cell cultures. For example, in 2019, Di Vito et al. evaluated the effects of a new medium formulation, EHFM (enriched Ham’s F12 medium), supplemented with 10% FBS, heparin 0.5 U/mL, epidermal growth factor (EGF) 50 ng/mL, FGF 25 ng/mL and bovine serum albumin (BSA) 1% with respect to the commonly used α-MEM and DMEM media, both supplemented with 10% fetal bovine serum (FBS) [41]. Cells maintained in EHFM showed higher proliferation rates compared with those grown in DMEM or α-MEM and a more marked alkaline phosphatase activity when cultivated in osteogenic medium (containing sodium β-glycerophosphate, ascorbic acid, and dexamethasone), suggesting a higher osteogenic potential. However, at the later stage of osteogenic differentiation, no calcium deposits were detected in hPDLSCs cultured in EHFM-differentiation medium. Mineralization was restored when EHFM-expanded hPDLSCs were transferred to a commercial culture medium for osteogenesis, even at higher levels compared to α-MEM- and DMEM-expanded hPDLSCs. Therefore, EHFM has shown to be a good medium formulation alternative for growth and stemness maintenance; however, its effects on osteogenic potential need to be further investigated [41].

IGF-1, recognized as a key protein in the development and growth of many tissues, has a role in regulating cell growth and differentiation of hPDLSCs. A study from Yan Y. et al. (2012) demonstrated that IGF-1 at the optimal concentration of 100 ng/mL was able to stimulate the in vitro proliferation ability of hPDLSCs as well as their osteogenic potential via ERK and mitogen-activated protein kinase (MAPK)/c-Jun N-terminal kinase (JNK) pathways. These effects were more evident when IGF-1-treated implants were transplanted in vivo, showing higher tissue mineralization compared to the control group [40]. In addition, combining 100 ng/mL IGF-1 with fibrin and cytokines in platelet-rich fibrin (PRF) significantly promoted the growth and proliferation of hPDLSCs in vitro. This combined treatment seemed to trigger the early osteogenic program in undifferentiated cells via the activation of the MAPK signaling pathway and phosphorylation of JNK [42].

Interestingly, the administration of FGF-2 (10 μg/mL), IGF-1 (10 μg/mL) and EGF (10 μg/mL) growth factors in combination with NGF (10 μg/mL) promoted neuronal differentiation of PDLSCs isolated from a beagle dog. However, PDLSCs treated with this cytokines’ mix showed high proliferation ability from the third day to the seventh day of culture [43]. This result confirmed the ability of FGF-2, EGF, IGF-1 and NGF to stimulate cell proliferation, suggesting the central role of cytokines’ mix for the ex-vivo expansion of hPDLSCs. 

Among the 11 structurally related proteins included in EGF family of ligands, Neuregulin-1 (Nrg-1) plays a key role in developing the adult nervous system, especially in neuron migration, nerve differentiation and myelination during both physiological and pathological processes. Recently, the role of NRG-1 in the proliferation, migration and angiogenesis of hPDLSCs has been described [44]. NRG-1 stimulated the expression of VEGF, platelet/endothelial cell adhesion molecule-1 (CD31) and hypoxia-inducible factor (HIF) in a dose-dependent manner. However, NRG-1 did not improve osteogenesis [44].

Erythropoietin (EPO) is a glycoprotein widely recognized for its role in supporting the survival of erythroid progenitor cells and stimulating their proliferation and differentiation [72]. Among the several biological functions exerted outside the hematopoietic system, the ability to stimulate osteoblastic differentiation and increase bone mass has been well described in the last decade [73]. Similarly, a direct action on hPDLSCs has been recently reported by Zheng DH et al. Their results showed that EPO, in the range of 5 to 20 U/mL, increased both the growth and the osteogenic differentiation of hPDLSCs in a time-and dose-dependent manner. Conversely, a high dose of EPO (50 U/mL) resulted in a slight decrease in cell viability and an increase in osteogenic differentiation [45,46]. Moreover, the Wnt/β-catenin signaling pathway has been suggested as a key pathway mediating EPO action on osteogenesis [46]. Interestingly, EPO doses < 20 U/mL also seem to exert a protective role in high glucose-induced oxidative stress and inhibition of both proliferation and osteogenic differentiation, a condition reported in diabetes. For this reason, it has been suggested as a novel adjuvant in therapy of diabetic periodontitis. Further studies aimed at characterizing the molecular mechanisms activated by the EPO could also suggest its use for the in vitro expansion of hPDLSCs. 

The most advanced stem cell-based tissue engineering aims at restoring periodontium via three key events: the reduction of the bacterial invasion, the controlled drug delivery inside the periodontal pocket and the endogenous MSC homing to the site of injury. Afterward, a balanced proliferation and osteogenic differentiation of MSC should be ensured. Among the factors involved in cell trafficking, the stromal cell-derived factor-1 (SDF-1) is a chemokine widely recognized for its role in bone marrow mesenchymal stem cell recruitment and tissue regeneration. A role for SDF-1 has also been reported in the subpopulation of hPDLSCs characterized by the coexpression of the STRO-1 antigen and CXCR4 receptor. In particular, the stimulation with SDF-1 at concentrations between 100 and 400 ng/mL significantly increased proliferation and migration of hPDLSCs. These effects were antagonized by a CXCR4-neutralizing antibody. On the other hand, SDF-1 promoted a small increase in alkaline phosphatase levels, negatively influencing the differentiation of these stem cell subpopulations [47].

Interestingly, when SDF-1α (200 ng/mL) was combined with PTH (50 ng/mL), proliferation, migration and osteogenic differentiation of hPDLSCs in vitro were significantly increased. Based on this evidence, the PTH/SDF-1α cotherapy protocol has been suggested for the reconstruction of destroyed periodontium [48].

### 2.2. Chemicals

Different drugs, commonly used to treat dysmetabolic syndrome, have been employed in the last decade to stimulate hPDLSC ex vivo expansion. The chemicals used to enhance hPDLSC expansion are listed in Table 1.

Statins represent one of the first drug groups to be investigated for the action on hPDLSC biology. Statins are inhibitors of 3-hydroxy-3-methylglutaryl coenzyme A reductase and are recognized as a first-line treatment against hypercholesteremia and as a preventative treatment for cardiovascular alterations [74,75]. Given that Simvastatin, a lactone form of statin found to be able to stimulate bone formation via the activation of the BMP-2 pathway, Zhao and Liu in 2013 investigated the effects of the statin on hPDLSC proliferation, osteogenic differentiation in vitro and tissue formation capacity in vivo [49,76]. They showed that high doses of Simvastatin inhibited hPDLSC proliferation, while low doses (0.01 or 0.1 μM) positively impacted both proliferation and osteogenic differentiation of the cells. hPDLSCs treated with low doses of simvastatin showed increased osteogenesis in vivo [49]. Therefore, Simvastatin has been suggested as useful for regenerative therapy due to its pro-osteogenic action; however, the dose-dependent effects on proliferation have discouraged its use for ex vivo expansion of hPDLSCs. 

The association between type 2 diabetes mellitus and periodontitis prevalence has been well established [50]. Metformin, recognized as the first-line oral medication for type 2 diabetes mellitus, is able to modulate hPDLSC behavior. In particular, metformin dose-dependently increased the proliferation, migration and osteogenic differentiation of hPDLSCs with the strongest effects reported after exposure to 50 mM metformin for 7 days [50]. Metformin also protects hPDLSCs against oxidative stress-induced damage via the activation of the Akt/Nrf2 signaling pathway [51].

In the last decade, major new advances have suggested that the Rho GTPases and downstream effectors such as Rho kinases play key roles in hPDLSC self-renewal, differentiation, adhesion, migration and apoptosis via direct modulation of the cytoskeleton [77,78]. In vitro treatment with Y-27632 (10 and 20 μM), a Rho-associated kinase (ROCK) inhibitor, enhanced the proliferation and migration of hPDLSCs and had no effect on apoptosis. This effect would appear to be relevant because cell migration is a key event during the repair and regeneration process. Normally, hPDLSCs migrate into injured sites and differentiate into local components such as fibroblasts, osteoblasts and cementoblasts participating in periodontal regeneration. However, Y-27632 inhibited ALP activity as well as mineral deposition and decreased osteogenesis-related gene expression levels of hPDLSCs. Furthermore, Y-27632 accounted for a switch from osteogenic to adipogenic differentiation. Although Y-27632 supplementation promoted hPDLSC proliferation and migration and maintained the stem cell properties, its inhibition of osteoblastic differentiation represents a major deterrent to hPDLSC preconditioning for regenerative therapy [52].

Other synthetic compounds are gaining ground as potential supplements to be added to culture media for ex vivo expansion of hPDLSCs for regenerative therapy. 

For example, Methylsulfonylmethane (MSM) is a DMSO derivative with relevant biological properties including antioxidant and anti-inflammatory effects. Treatment with increasing doses of MSM (1 mM, 5 mM, 10 mM and 25 mM) for 7 and 21 days accounted for a slight increase in the proliferation rate of hPDLSCs compared with untreated cells, with the highest level of cell proliferation reported after exposure to 10 mM MSM. In contrast, a higher MSM dose (50 mM) showed a cytotoxic effect. Interestingly, this sulfur compound proved to be a potent inducer of osteoblastic differentiation of hPDLSCs in vitro and in vivo. A dose-dependent increase of in vitro mineralization has been reported in hPDLSCs after exposure to 5 mM and 10 mM MSM. Furthermore, MSM was able to support hPDLSC differentiation into osteoblasts after transplantation in an in vivo calvarial defect model. Osteogenic action seems to be mediated by the stimulation Smad2/3 signaling pathway upstream of RUNX2, the master gene associated with osteoblast differentiation. These results configure MSM as a good candidate for future clinical applications in alveolar bone regeneration [53].

Sulfonated chitosan oligosaccharide (SCOS), a heparan-like compound prepared from the deacetylation and hydrolysis of chitin, also seems to play a predominant role in hPDLSC osteoblastic differentiation. Given the affinity between SCOS and FGF-2, observed via surface plasmon resonance analysis, the effects produced by SCOS on cultured hPDLSCs were analyzed. SCOS alone had no significant effect on proliferation compared to bFGF (20 ng/mL); however, when hPDLSCs were treated with combined SCOS and bFGF at different proportions (1:1, 1:5, and 1:10), a strong proliferative advantage was observed, especially at the molar ratio bFGF:SCOS (1:1). In addition, SCOS was able to reduce the inhibitory effect of 20 ng/mL FGF-2 on the osteogenic differentiation of hPDLSCs in vitro [54].

Potassium dihydrogen phosphate (KH2PO4; 1.8 mmol/L) was recently recognized as an attractive potassium salt with a marked ability to induce the proliferation and odonto/osteogenic differentiation of hPDLSCs via the NF-κB pathway. The treatment with BMS345541, a specific NF-κB inhibitor, was able to attenuate odonto/osteogenic differentiation of KH2PO4-treated hPDLSCs. These studies suggested the potential role of potassium dihydrogen phosphate in the formation of periodontal tissues; therefore, its use could be introduced into clinical practice for regenerative medicine [55].

The recombinant COMP-Ang1 is a therapeutic agent that has received much attention for its ability to stimulate angiogenesis similarly to Ang-1, without presenting the management difficulties of Ang-1. It is a chimera from angiopoietin-1 (Ang1) and a short coiled-coil domain of cartilage oligomeric matrix protein (COMP). The treatment of bone marrow MSCs with increasing doses (300 and 600 ng/mL) of COMP-Ang1 accounted for increased proliferation, homing and expression of Runx2 by activating the Tie-2/Angiopoietin pathway. Its activity was abrogated when Tie-2 was knockdowned, preventing the phosphorylation of p38 MAPK and Akt, or when cells were treated with LY294002 and SB203580, inhibitors of phosphoinositide 3-kinase (PI3K) and p38 MAPK, respectively [56]. The effects of COMP-Ang-1 described in bone marrow MSC cultures suggest a similar potential effect on hPDLSCs; however, further studies are needed to clarify this aspect.

### 2.3. Natural Compounds

Many natural compounds have been reported to modulate MSC proliferation, the main ones of which are listed in Table 1. Recently, it was demonstrated that melatonin (MLT) supplementation in complete culture medium (α-MEM 10% FBS) ameliorated hPDLSC long-term expansion-caused cellular senescence by restoring autophagy [57]. In particular, the treatment of P7 and P15 cells with 0, 10 nM, 100 nM, 1 μM, or 10 μM MLT for 24 h induced cell rejuvenation by promoting the autophagic processes via the PI3K/AKT/mTOR signaling pathway in a MLT receptor-dependent manner. Rescue experiments using the autophagy inhibitor 3-MA blocked MLT-induced cell rejuvenation, confirming autophagic process modulation by MLT. In the future, the treatment of hPDLSCs with autophagy-restoring agents could be effective in developing large-scale cell production protocols for cellular therapy and regenerative medicine [57]. 

In the panel of natural compounds, a significative role is played by Progranulin (PGRN), a multifunctional protein known for its ability to suppress TNF-α-mediated inflammation via the antagonism of TNFRs [58]. It plays a central role in the maintenance of numerous tissues, exerting important action on neurons, epithelial cells and immune cells. The addition of PGRN to culture medium at the optimal concentration of 25 ng/mL induces the proliferation of hPDLSCs in vitro. In addition, PGRN was able to reverse TNF-α-mediated inhibition of extracellular matrix calcification in both inflammatory and noninflammatory conditions. Unfortunately, PGRN has not yet been tested in animal models to provide preclinical evidence as a promising treatment for periodontitis [58].

Rutin is another citrus flavonoid glycoside present in many plants and is considered one of the best natural antioxidants. Recently, its impact on hPDLSCs has been described; it promoted cell proliferation and osteoblastic differentiation at the optimal concentration of 1 × 10^−6^ mol/L, especially when the differentiative medium was supplemented with 20 µg/mL vitamin C [59].

Similarly, Nicotinamide treatment (0 μM, 50 μM, 100 μM, 200 μM and 300 μM) for 8 days was revealed to be effective in inducing hPDLSC proliferation and differentiation. This water-soluble form of vitamin B3 exerts its molecular activity by inducing miR-22-3p-mediated silencing of SIRT1. As expected, miR-22-3p knockdown blocked the self-renewal and differentiation ability of hPDLSCs [60].

Naringenin (NAR) is a natural citrus flavonoid recognized for its anti-inflammatory, antioxidant, and anticarcinogenic activities. This compound has been shown to be effective in improving the number and bioactive function of hPDLSCs in the PDL. In vitro, the addition of NAR to standard MSCs culture media at a concentration of 1 μM promoted a proliferation effect, while at 10 μM sustained osteogenic and endothelial differentiation. For these reasons, it could have clinical importance for the regeneration and repair of alveolar bone [61].

Phyto derivatives from the citrus family seem to be particularly effective for hPDLSC ex vivo expansion. Indeed, n-hexane, methylene chloride (MC), ethyl acetate (EA), n-butanol (BuOH) and four fractions from Z. Schinifolium significantly enhanced in vitro proliferation and osteogenic potential of hPDLSCs in a dose-dependent manner [62]. Myricetin and Baicalein, two other compounds isolated mainly from plants, were tested on hPDLSCs in order to understand if their supplementation in culture media could favor the expansion and osteoblastic differentiation of these immature cells. 

Myricetin is a flavonoid commonly found in tea, berries, onions, herbs and red grapes. Its pharmacological effects, including antimicrobial, antioxidant and anticancer activities, are well demonstrated [71]. In vitro exposure of hPDLSCs to Myricetin concentrations up to 1 μM for 24 h and 48 h resulted in a moderate increase of cell proliferation in a time-dependent manner, while higher concentrations caused cytotoxicity. Interestingly, myricetin treatment for 7 days and 14 days significantly promoted osteogenic differentiation via the regulation of the BMP-2/Smad and ERK/JNK/p38 pathways [63].

Baicalein is instead a polyphenolic flavonoid compound extracted from the roots of Scutellaria baicalensis and Scutellaria lateriflora. Historically, it boasts antioxidant, antivirus, antibacterial, anti-inflammatory and anti-allergic properties. Given its heterogeneous properties, the potential therapeutic effects of Baicalein on hPDLSCs were evaluated. In vitro evidence was provided that Baicalein promoted hPDLSC osteogenesis in a dose-dependent manner (1.25–10 μM), especially when cells pretreated with Baicalein (20, 40 or 80 μM) were then stimulated with LPS (200 ng/mL) for 2 h. Moreover, Baicalein suppressed the inflammatory response, functioning as a Wnt/β-catenin signaling activator [64,65].

Surely, Myricetin and Baicalein showed a positive effect on the differentiation capacity of hPDLSCs towards the osteoblastic lineage; however, as expected, they did not significantly promote the ex vivo expansion of these mesenchymal-like cells. In addition, the stemness properties remain to be investigated.

Interestingly, the oxytocin receptor was expressed on hPDLSC surface. The treatment with 50 nM of oxytocin significantly increased hPDLSC expansion as well as upregulated expression of osteogenesis-related genes in comparison to osteogenic inductive medium without oxytocin. In this in vitro cellular model, oxytocin promoted the mineralized nodule formation, stimulating the phosphorylation of the ERK and protein kinase B (AKT) pathways and inhibiting phosphorylation of the PI3K pathway [66].

### 2.4. Extracellular Matrix (ECM)

An intriguing approach to overcome problems related to long-term in vitro culture requires the seeding of hPDLSCs on a self-derived decellularized extracellular matrix (ECM) of hBMSCs. Autologous ECM seems to account for hPDLSC attachment, ex vivo expansion and differentiation potential while maintaining stemness properties. 

However, ECM of hBMSCs is obtained through invasive, complex and painful procedures and the results are limited by its source and quantity [79,80]. In light of these critical issues, the growth of hPDLSCs on ECM from hBMSC could be poorly applicable.

A turning point in ECM-based MSC expansion was suggested in 2008 by Zhang et al., who first isolated human urine-derived stem cells (hUSCs). hUSCs boast the same properties of BMSCs, including clonogenicity, self-renewal and multidifferentiation potential, and can be collected noninvasively and at low cost, making them an adequate and efficient source of ECMs [81].

Recently, Xiong et al. investigated the influence of ECM deposited by human urine-derived stem cells (UECM) on hPDLSCs, in vitro and in vivo, comparing it to ECM deposited by hPDLSCs (PECM) and tissue culture plate coated by fibronectin (Table 1) [67]. The results were very interesting: both UECM and PECM markedly promoted hPDLSC proliferation; however, UECM accounted for osteogenesis and angiogenesis to a greater extent than PECM. On the other hand, PECM sustained the adipogenic differentiation of hPDLSCs. Fibronectin-coated tissue culture dish stimulated the adhesion and spreading of hPDLSCs to a greater degree than UECM and PECM but, unfortunately, was not able to promote osteogenesis. Although UECM is characterized by dense bundles of fibers containing abundant fibronectin, the performance of fibronectin coating was significantly lower than that of UECM [67].

Undoubtedly, Xiong et al. have emphasized the value of hUSCs in regenerative periodontal therapy. The extremely low cost-benefit ratio could candidate hUSCs for the industrial production of ECMs, and, thus, obtain biological scaffolds with many desirable properties, including biocompatibility, bioactivity and biosafety [67].

**Figure 1:**
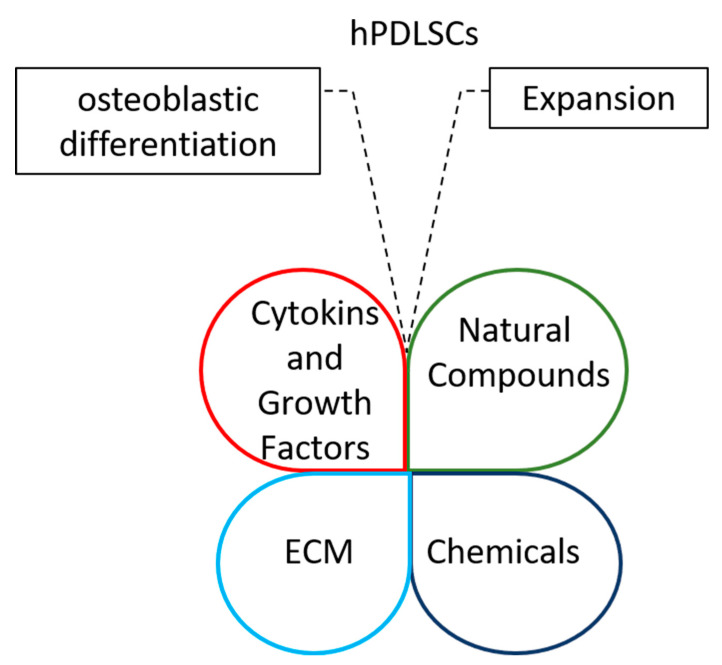
Diagrams highlight the opportunity of integrating cytokines and growth factors, chemicals, natural compounds and ECMs to improve the expansion of periodontal ligament stem cells and their osteoblastic differentiation capacity.

Interestingly, cytokines and growth factors, chemicals, natural compounds and ECM could be combined to improve stemness and osteoblastic potential of periodontal ligament stem cells for regenerative therapy (Figure 1).

## 3. Human Blood Derivatives and Allogenic Serum Supplements as New Frontiers in In Vitro hPDLSC Expansion

The quality and safety of hPDLSCs for clinical applications could be compromised by their exposition to animal proteins during cell expansion. Although FBS is widely accepted as a standard nutritional supplement, its use in clinical trials is discouraged by regulatory authorities due to the serious adverse effects that could be generated by its xenogenic origin, including infectious disease transmission and immunological reactions [82].

**Figure 2:**
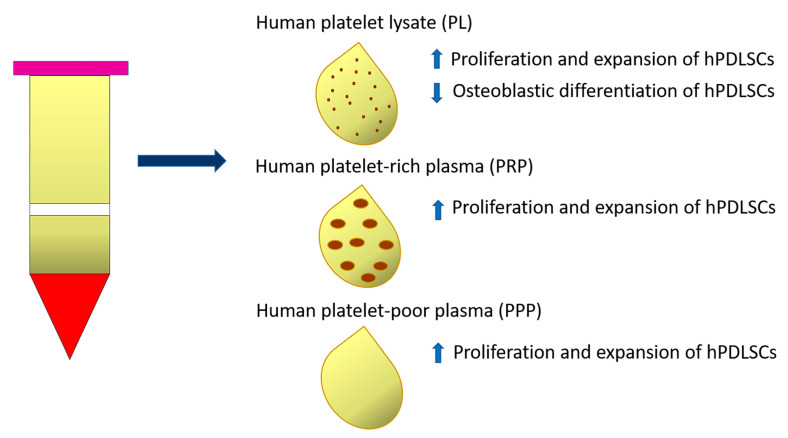
Diagram representing the effect of Human platelet lysate (PL), Human platelet-rich plasma (PRP) and Human platelet-poor plasma (PPP) on proliferation and expansion of PDLSCs and their osteoblastic differentiation ability.

Human platelet-rich preparations, and, notably, human platelet lysate (PL), or autologous serums have been suggested as FBS alternatives for the clinical scale manufacture of cells (Table 1). In particular, 10% PL (isolated from 20 donors aged 18–35 years) added as adjuvant in culture media (α-MEM) supported the proliferation and expansion of either young (19–25 years) or old (48–63 years) hPDLSCs to the same extent as FBS, without alterations of their immunomodulatory properties [80]. The effects of PL supplementation on hPDLSC expansion are attributable to a cocktail of growth factors, such as platelet-derived growth factor (PDGF), insulin-like growth factor (IGF), basic fibroblast growth factor (bFGF) and transforming growth factor-β1 (TGFβ1) present in this type of human blood-derived product. The neutralization of FGF alone or in combination with PDGF significantly decreased the proliferative effect of PL on hPDLSCs. However, PL affected in vitro differentiation of hPDLSCs isolated from either ‘young’ or ‘old’ donors, by decreasing ALP activity and osteogenic gene expression. The osteogenic potential of PL-expanded hPDLSCs was restored by further incubating cells in osteoinductive media containing FBS. The PL-based expansion protocol represents a valid alternative to FBS supplementation and in the future could be implemented with molecules able to preserve the osteogenic potential of hPDLSCs [68] (Figure 2).

Another study demonstrated the effect of two autologous platelet-derived fractions, platelet-rich plasma (PRP) and platelet-poor plasma (PPP), on hPDLSCs, comparing them to FBS. Specifically, PRP and PPP were derived from three healthy male volunteers who did not match to periodontal ligament donors. Both PRP and PPP selectively stimulated hPDLSC proliferation and clonogenic ability when compared to FBS after 2 days of stimulation, especially in the cell subpopulation positive for CD73 and CD90 cell surface markers. Otherwise, no significant differences were observed regarding the proliferation of CD146- or CD105-positive cells when stimulated with PRP, PPP or FBS [69] (Figure 2).

Although these findings identify PRP and PPP as safer sources of growth factors for isolation and expansion of periodontal cells suitable for periodontal regeneration, further studies are needed to understand the impact of PRP and PPP on hPDLSC osteogenic potential [69].

Afterward, Arpornmaeklong et al. examined how DMEM-F12 culture medium supplemented with four different types of 10% serums, fetal bovine serum (FBS), allogeneic human male AB serum (HS), in-house autologous (Auto-HS) and in-house allogeneic human serums (Allo-HS), modulated hPDLSC properties. Specifically, during short-term expansion (until passage 5), HS promoted growth and osteogenic differentiation of hPDLSCs compared to FBS; however, these effects decreased in the expanded hPDLSCs (passage 15). The growth rate and osteoblastic differentiation ability of hPDLSCs in Auto-HS and Allo-HS were comparable to FBS [82]. Allogeneic human serum supplement represents a great alternative to FBS for hPDLSC culture as it allows overcoming the limitations related to the autologous serum, including limited availability, reduced quality of serum associated to advanced age and medical conditions. Allogeneic human serum has the advantage of being a highly biocompatible product enriched in growth factors (PDGF, EGF and b FGF) essential for angiogenesis, proliferation and differentiation of MSCs and increases accessibility to stem cell transplantation for everyone [70,83].

The development of new xenofree media is essential to expand hPDLSC-based therapeutic approaches via the preservation of both their differential mesengenic potential and genomic stability [84].

## 4. Conclusions

hPDLSCs represent an attractive source of MSCs from the oral cavity for periodontal tissue regeneration. MSCs from PDL show great therapeutic potential due to their capacity for self-renewal, multilineage differentiation and low immunogenicity. Despite this, hPDLSC-based therapies are limited by the small number of cells obtained after isolation and by the common long-term expansion methods often associated with the reduction of stemness, differentiation potential and, in some cases, even senescence in vitro.

To date, new culture media, chemicals and natural compounds have been proposed in order to expand hPDLSCs on a large scale and increase their biological functions (Table 1). 

Although the evidence discussed here represents a great advance in the field of hPDLSC expansion, many studies still need to be carried out primarily to overcome problems related to the use of animal products such as FBS. The supplementation of culture media with FBS is still widely used to support the growth of several cells in vitro; however, it could induce hyperimmunogenicity, causing cell therapy rejection and possible infections. For these reasons, new hPDLSC expansion protocols based on human serums or PLs, or PRP, are being tested.

The combined application of human blood derivatives and molecules, sustaining self-renewal and differentiation capabilities, could be an advantageous strategy for reinforcing proliferation, stemness, osteoblast production and cytokine secretion of hPDLSCs.

## Figures and Tables

**Table 1:** Schematic representation of the effects of new cell culture media formulations on stemness (CD73, CD90, CD105), expansion (number of actively dividing cells) and osteoblastic differentiation (ALP, osteocalcin, osteopontin, calcium deposits) of PDLSCs in vitro. Green and red indicate, respectively, the positive and negative effects exerted by each culture medium on multipotent stem cells isolated from PDL, and orange indicates that a specific type of supplementation has no activity on the stem or differentiation properties of PDLSCs. If the test reagent has not yet been investigated for specific biological properties of PDLSCs, it is indicated with a light-yellow color.

Compounds Supplemented to Culture Media for Healthy PDLSCs		Stemness	Expansion	Osteoblastic Differentation	Ref.
Cytokins and Growth Factors	FGF2 (10 ng/mL) and A83-01 (5 μM)				Zhang et al., 2019 [37]
	VEGF (25 ng/mL)				Lee et al., 2012 [38]
	bFGF (25 ng/mL) + BMP-2 (50 ng/mL)				Kang et al., 2019 [39]
	IGF (100 ng/mL)				Yan et al., 2012 [40]
	EHFM medium				Di Vito et al., 2019 [41]
	Platelet-rich fibrin (PRF) + IGF-1 (100 ng/mL)				Li et al., 2018 [42]
	b-FGF-2 (10 μg/mL) + IGF-1 (10 μg/mL) + EGF (10 μg/mL) + NGF (10 μg/mL)				Li et al., 2020 [43]
	Neuregulin-1 (0, 5, 10, and 20 ng/mL)				Li et al., 2022 [44]
	Erythropoietin (0, 5, 10, 20, 50 U/mL)				Zheng., et al. 2019 [45,46]
	SDF-1 (100 and 400 ng/mL)				Du et al., 2012 [47]
	SDF-1α (200 ng/mL) + PTH (50 ng/mL)				Du et al., 2016 [48]
Chemicals	Simvastatin (0.01 or 0.1μM)				Zhao and Liu et al., 2013 [49]
	Metformin (50 nM)				Sanz et al., 2018 Jia et al., 2020 [50,51]
	Y-27632 (10 and 20 μM)				Wang et al., 2017 [52]
	Methylsulfonylmethane (1, 5, 10, 25 or 50 mM)				Ha et al., 2020 [53]
	Sulfonated chitosan oligosaccharide (SCOS) + bFGF (20 ng/mL): molar ratios: 1:1; 1:5; 1:10)				Li et al., 2020 [54]
	Potassium dihydrogen phosphate (1.8 mmol/L)				Xu et al., 2019 [55]
	COMP-Ang1 (300 and 600 ng/mL)				Kook et al., 2014 [56]
Natural Compounds	Melatonin				Tan et al., 2021 [57]
	Progranulin (25 ng/mL)				Yu et al., 2021 [58]
	Rutin (1 × 10^−6^ mol/L) + vitamin C (20 µg/mL)				Zhao et al., 2019 [59]
	Nicotinamide (0 μM, 50 μM, 100 μM, 200 μM and 300 Μm)				Zheng et al., 2020 [60]
	Naringenin (1–10 uM)				Zhang et al., 2021 [61]
	*Z. schinifolium* (5, 10, 25, 50, 100 μg/mL)				Kim et al., 2015 [62]
	Myricetin (0.01, 0.1, and 1 μM)				Kim et al., 2018 [63]
	Baicalein (1.25–10 μM)				Chen et al., 2017 [64]
	Baicalein (20, 40, or 80 μM) + LPS (200 ng/mL)				Ren et al., 2021 [65]
	Oxytocin (50 nM)				Ge et al., 2019 [66]
ECM	UECM (human urine-derived stem cells)				Xiong et al., 2019 [67]
Human Serums	Human platelet lysate (PL)				Wu et al., 2017 [68]
	Autologous platelet-rich plasma (PRP) and platelet-poor plasma (PPP)				Martínez et al., 2019 [69]
	House allogeneic human serums (Allo-HS)				Arpornmaeklong et al., 2018 [70]

## Data Availability

No new data: Data sharing not applicable to this article as no datasets were generated or analyzed during the current study.
